# Comparing Stroop-like and Simon Effects on Perceptual Features

**DOI:** 10.1038/s41598-017-18185-1

**Published:** 2017-12-19

**Authors:** Elisa Scerrati, Luisa Lugli, Roberto Nicoletti, Carlo Umiltà

**Affiliations:** 1 Department of Philosophy and Communication, University of Bologna, Bologna, Italy; 2 Department of General Psychology, University of Padua, Padua, Italy

## Abstract

Stroop-like and Simon tasks produce two sources of interference in human information processing. Despite being logically similar, it is still debated whether the conflicts ensuing from the two tasks are resolved by the same or different mechanisms. In the present study, we compare two accounts of the Stroop-like effect. According to the Perceptual Account, the Stroop-like effect is due to Stimulus-Stimulus congruence. According to the Decisional Account, the Stroop-like effect results from the same mechanisms that produce the Simon effect, that is, Stimulus-Response compatibility. In two experiments we produced Stroop-like and Simon effects by presenting left/right-located stimuli consisting of a colored square surrounded by a frame of the same color as the square or of a different color. Results showed that discriminating either the color of the square (Experiment 1) or that of the frame (Experiment 2) yielded additive Stroop-like and Simon effects. In addition, the patterns of temporal distributions of the two effects were different. These results support the Perceptual Account of the Stroop-like effect and the notion that the Stroop-like effect and the Simon effect occur at different processing stages and are attributable to different mechanisms.

## Introduction

Imagine you are speaking at an important workshop over Skype and all of a sudden an alarm in your building sets off. What would you do, besides thinking that this is the weirdest Skype call you have ever been on? The difficulty to act in such a situation lies in the fact that our information processing system must pay selective attention to the primary task while trying to ignore the co-occurring stimulus. Our lives are filled with such (maybe less theatrical) situations.

In this article, we attempt to investigate how selective attention works by making recourse to the factorial combination of two well established cognitive tasks, that is, the Stroop-like task and the Simon task. These tasks are seen as gaining access to the basics of cognition, providing clues to the fundamental process of attention. In doing so we will make reference to the Dimensional Overlap model (DO) and taxonomy developed by Kornblum and his colleagues^1–5^.

In Kornblum’s taxonomy, eight types of compatibility tasks are distinguished and each is assumed to give rise to a specific type of compatibility effect. The core idea of this taxonomy is that of *dimensional overlap*, that is, “the degree to which a stimulus set and a response set, or two or more aspects of a stimulus set or a response set, are perceptually, structurally, or conceptually similar”^3^ (p. 875). In the present study, we are concerned with the Type-3 and Type-4 effects. The former is also known as the “Simon effect”^6–10^ and is characterized by a dimensional overlap between the response set and a task-irrelevant stimulus dimension. The Type-4 effect, also known as “Stroop-like effect”^11–14^, is characterized by a dimensional overlap between two stimulus dimensions, one relevant for performing the task and the other non-relevant. The Stroop-like effect is often referred to as S-S overlap. However, in the present paper we will be using the term Stroop-like in accordance with Kornblum’s taxonomy.

In tasks that produce the Simon effect, that is, in “Simon tasks”, the stimulus spatial position is not task relevant and participants respond to a non-spatial stimulus attribute (e.g., color, shape or pitch). The effect emerges because responses are faster and more accurate when stimulus position (i.e., an irrelevant stimulus dimension) and response position (i.e., a relevant response dimension) correspond (e.g., right stimulus–right response) than when they do not (e.g., right stimulus–left response). Several authors posit that the Simon effect results from the automatic coding of stimulus position, which, in turn, automatically activates the spatially corresponding response, thus producing a competition, at the response selection stage, between the spatially corresponding response and the response required on the basis of task instructions^2,5^ (though, see^15^ for a different explanation of the Simon effect; see^10,16,17^ for reviews).

In the classical Stroop task^14^, participants respond to the ink color of stimuli consisting of words denoting colors. Responses are faster when the relevant stimulus dimension (i.e., the ink color) and the irrelevant stimulus dimensions (i.e., the name of the color) are congruent (e.g., the word “red” printed in red) rather than incongruent (e.g., the word “red” printed in green). In Stroop-like tasks, such as the flanker task^11^, the target (e.g., the letter F or G) represents the relevant stimulus to which participants have to respond, while different distractor objects, the flankers, convey the irrelevant stimulus information. Responses are faster and more accurate when target and flankers are congruent (i.e., the central target letter “G” sided by the flanker letters “G” as in “G G G”) rather than incongruent (i.e., the central target letter “G” sided by the flanker letters “F” as in “F G F”). Despite being conceptually similar to the classical Stroop task, Stroop-like tasks often do not replicate Stroop’s original task. This is because the classical Stroop task also involves stimulus-response overlap in addition to stimulus-stimulus overlap. Indeed, the original version of the Stroop task required participants to respond vocally to the ink color of the stimulus (i.e., a word denoting a color), so that a response dimension overlap occurred with both the relevant (i.e., the ink color) and the irrelevant stimulus dimensions (i.e., the meaning of the word denoting the color). For this reason, in Kornblum’s latest taxonomy^4^, the “Stroop effect”, which is produced by the classical Stroop task, is a Type-8 effect. Therefore, the term Type-4 effect, which is defined by a sole S-S overlap, only applies to those versions of the Stroop task in which subjects respond manually.

An issue that has attracted much interest is whether the classical Stroop and Stroop-like effects occur at the same processing stage as the Simon effect (for example, see^18,19^). This issue can be examined by creating a combination of tasks and testing for an interaction between the compatibility effects^20–22^. If the compatibility effects do not interact (i.e., they are additive) one can conclude with reasonable certainty that the conflicts are independent and are likely mediated by different mechanisms (see^23^ for a review; see below for a more extensive discussion of the additive factor method, AFM, introduced by Sternberg^24^). For example, a factorial task-crossing design would be one that combines Simon and Stroop tasks by presenting color-word stimuli at peripheral locations. Although the results of these studies are not crystal-clear (e.g., in the case of Hommel’s study^20^, two out of three experiments showed interactivity) the accepted conclusion is that they attest to additivity. That is, the conflicts that characterize Stroop-like and Simon tasks would occur at different processing stages.

The issue of whether Stroop-like and Simon effects occur at the same or at a different processing stage, and, thus, are mediated by the same mechanism or by different mechanisms, has also been explored by making recourse to the sequence congruency effect. Gratton, Coles, and Donchin^25^ were the first to observe a sequence congruency effect, that is, an interaction between previous and current trial congruency, according to which congruency effects were smaller following incongruent trials than following congruent ones (also, see^23,26^ for reviews). This phenomenon is often explained on the basis of the conflict monitoring model, which holds that the congruency sequence effect stems from conflict-driven adjustments in cognitive control^23,27^ (though alternative explanations were put forward^28–30^; see^23,26^ for reviews). A strategy for exploring whether Stroop-like and Simon effects occur at different processing stages and depend on different mechanisms consists of constructing an experiment that combines the two tasks while comparing sequence congruency effects within tasks and between tasks^23,26^. If the two effects involve the same mechanisms, the sequence congruency effect should be the same between and within tasks. If the mechanisms are different, there should be no sequence congruency effect between tasks. A number of studies have adopted this strategy^31–37^. Based on the results of these studies, a fairly good case can be made for the claim that conflict resolution in Stroop-like tasks is mediated by a different process (and associated neural circuitry) than in Simon tasks^32^. Specifically, conflict resolution in the Stroop task likely involves the excitatory biasing of task-relevant stimulus processing^32,38^, whereas conflict resolution in the Simon task likely involves the inhibition of direct route response-priming processes^32,39^.

Having reached this stage, one might conclude that the issue is settled and no further evidence is needed: Stroop-like effects and the Simon effect occur at different processing stages and involve different mechanisms. However, a closer look at the available evidence suggests that further inquiry might be useful. Stimuli in Stroop tasks, but also in Stroop-like tasks, are very often verbal in nature. There is, therefore, the possibility that the lack of an interaction between Simon and Stroop-like effects is attributable to the different nature of the stimuli rather than to the two effects being mediated by different mechanisms. As will be discussed in more detail below, while interference in the classical Stroop task originates from a semantic conflict between ink color and meaning of the verbal stimulus, interference in the Simon task originates from a non-semantic conflict between the different locations occupied by stimuli and responses.

Let us now take into consideration the reason why the mechanisms should be different. Because the stimuli in the Stroop-like task (i.e., colored words denoting colors in the manual Stroop task and letters in the flanker task) are unrelated to the responses (i.e., left-right keypresses), congruence effects might be attributable to processes occurring at the stage of stimulus identification rather than response selection, as, instead is the case for the Simon task. That is, given that the only overlap in the Stroop-like task is that between the relevant stimulus dimension and the irrelevant stimulus dimension (i.e., the ink color and the name of the color in the manual Stroop task; the target and flanker letters in the flanker task), neither response activation nor response competition processes, able to produce compatibility effects, would occur. This explanation, known as the Perceptual Account of the manual Stroop effect and the Stroop-like effect, is based on the presence or absence of S–S congruence^2,5^ (also, see^15^ for S-S congruence effects). As already mentioned, much evidence supporting the Perceptual Account of Stroop and Stroop-like effects, and thus for the independence of these effects from the Simon effect, has been gathered^18,20–22,40–42^. Basically, the main finding of these studies consists in showing additive rather than interactive Stroop and Simon effects. According to Sternberg’s^24^ AFM, if two factors do not interact, they are said to be additive and are thought to affect different processing stages. Additivity between two factors means that they operate at two different processing stages that take place serially (also, see^43^). In addition, evidence supporting the Perceptual Account of the Stroop-like effect has been reported by studies assessing conflict adaptation effects, that is, the reduction of interference arising when two consecutive incongruent stimuli occur in a sequence of mixed trials (also, see above). Conflict adaptation effects are specific to the type of conflict involved, thus suggesting that different types of conflict (i.e., Simon, Stroop) are resolved by different mechanisms at different processing stages^30,32,44,45^ (for reviews, see^23,26^). Moreover, it appears that spatial peripheral cues modulate the spatial Stroop effect but not the Simon effect^46,47^. Also, Correa, Cappucci, Nobre, and Lupianez^48^ found a smaller Stroop effect with targets appearing at a cued time windows rather than at an uncued one, whereas the Simon and flanker effects increased when the time windows was cued rather than uncued.

However, a different explanation, which we term the Decisional Account and is based on the notion of short-term stimulus-response associations, explains the Simon effect and might be invoked to explain also Stroop and Stroop-like effects. One has only to assume that short-term associations, created on the basis of task instructions (e.g., red stimulus–left keypress), can give rise to S-R congruence effects^49–51^ (for reviews see^52,53^). For instance, in the classical Stroop task, both the ink color of words denoting colors (i.e., the relevant stimulus dimension) and the name of the color (i.e., the irrelevant stimulus dimension) would activate the responses associated with them on the basis of task instruction. Similarly, in Stroop-like tasks such as the flanker task, both the target (i.e., the relevant stimulus dimension) and the flankers (i.e., the irrelevant stimulus dimension) would activate the responses associated with them on the basis of task instructions. As a consequence, on congruent trials both the relevant and the irrelevant stimulus dimensions would activate a common response code, whereas on incongruent trials the relevant and irrelevant stimulus dimensions would activate competing response codes. Therefore, according to the Decisional Account, both the classical Stroop and the Stroop-like effects would be originated by the same mechanisms involved in the Simon effect. Results compatible with the Decisional Account were provided by a number of studies showing either S-R compatibility underlying Stroop and Stroop-like effects^49^ or an interaction between Stroop-like and Simon effects^19,20,42,54^. As already noted, according to Sternberg’s^24^ AFM, if two factors interact, then they are thought to affect the same processing stage.

The aim of the present study is to shed light on these discrepant results. To this end, we contrasted the Perceptual Account and the Decisional Account by combining the Stroop-like effect with the Simon effect in a factorial design, while controlling for stimulus attributes. As was pointed out by Li, Nan, Wang, and Liu^18^, previous studies that investigated Simon and Stroop effects were not conclusive because of differences in stimulus attributes between the two tasks. Specifically, Li *et al*. argued that, while interference in the classical Stroop task stems from semantic conflict (i.e., ink color vs. meaning of the word), interference in the Simon task stems from a non-semantic conflict (i.e., different locations for stimulus and response). Therefore, it is uncertain whether the conflicts that cause the Stroop and Simon effects are distinct, as the Perceptual Account claims^2,5^, or mere differences in the nature of the stimuli (i.e., words vs. perceptual stimuli) are responsible for the observed dissimilarities. For example, Hommel^20^ (Experiment 1) asked participants to press a left or right key in response to the ink color of target words (i.e., names of colors), which were randomly presented on the left or right side of the screen. In such a paradigm, the Stroop conflict concerned the ink color (i.e., relevant stimulus dimension) and the meaning of the word (i.e., irrelevant stimulus dimension), whereas the Simon conflict concerned stimulus and response spatial positions. Results from this study showed additive Stroop and Simon effects (also see^22,41^). In addition, the RT distributions of the two effects were opposite: The Stroop effect increased, whereas the Simon effect decreased with increasing RTs. However, this outcome might simply reflect differences in the nature of the stimuli, because the two conflicts concerned different stimulus attributes (i.e., color vs. meaning for the Stroop effect, and color vs. spatial position for the Simon effect).

To avoid these shortcomings, Li *et al*.^18^ (Experiment 2) introduced a spatial-arrow Stroop task and combined it with a Simon task. In the former, the conflict concerned spatial information between arrow locations (i.e., top or bottom on the screen) and arrow orientation (i.e., upward or downward). In the latter, the conflict concerned locations of the arrows (i.e., left or right) and locations of the responses (i.e., left or right). Thus, conflicts resulting from either the spatial-arrow Stroop task or the Simon task were related to spatial attributes of the stimuli. Results showed that Stroop-like and Simon effects did not interact even though both originated from spatial attributes.

However, a criticism of the study by Li *et al*.^18^, and all previous studies using arrows^30,44,46–48^, is that the Stroop-like conflict was still somehow dependent on a semantic dimension. Indeed, the authors required participants to interpret/decode the direction of an arrow. This procedure might have induced some kind of semantic processing. Although words differ from arrows, given that the processes underlying the interpretation of words are more complex than those underlying the interpretation of arrows^55^, both types of stimuli pertain to a semantic typology (i.e., projective vs. deictic, see also^56^).

We, therefore, introduced a perceptual manipulation of the stimulus for either task. As in previous studies investigating Simon and Stroop-like effects^19,40^, we chose colored stimuli. Unlike those studies, however, we did not adopt a flanker paradigm. For instance, in the study by Treccani, Cubelli, Della Sala, and Umiltà^19^, participants had to judge the color of a central target that was presented together with a left or right-located colored flanker of the same color as the target or of a different color. Thus, the flanker conveyed both the irrelevant spatial information, which might or might not correspond to the response position and produces the Simon effect, and the irrelevant color information, which might or might not be congruent with the target color and produces the Stroop-like effect. Results showed an interaction between the two types of conflict, suggesting that the Simon effect and the Stroop-like effect (i.e., the flanker effect) are both ascribable to the same processing stage (i.e., the response selection or decisional stage). However, the authors acknowledged that their paradigm might have triggered perceptual grouping^57,58^, and referential coding^59,60^, which would be compatible with a Perceptual Account of the Stroop-like effect. In particular, they acknowledged that, when the target and the flanker were of the same color (i.e., congruent condition), they could be seen as forming a perceptual group, that is, one single object shifted to one side of the display (i.e., towards the flanker position). For example, a red target presented with a red flanker on the right could be seen as one red object shifted to the right. In contrast, when the target and the flanker were of different colors (i.e., incongruent condition), the flanker might have served as a reference point for the spatial coding of the target. For example, a red target presented with a green flanker on the right might be coded as left, given that it is on the left side of the flanker.

To avoid these confounds, we chose a different Stroop-like paradigm. Our stimuli consisted of colored squares surrounded by a frame that could be of the same color as the square or of a different color. Therefore, in our study the target contained both the task relevant and the task irrelevant information. In this way, we ruled out a possible influence of perceptual grouping or of referential coding, given that our stimuli were built such that the irrelevant stimulus dimension (i.e., the frame) encircles the target (i.e., the square) rather than flanking it. In our study, the Stroop-like conflict concerned the color of the square (i.e., red or blue) and the color of its frame (i.e., red or blue). The Simon conflict concerned the position of the stimulus (i.e., left or right) and the position of the response (i.e., left-right keys).

In sum, by following Li *et al*.^18^ we compared Stroop-like and Simon effects on a single dimension. That is, interference in either task stems from perceptual conflicts rather than from conflicting semantics (name of the color and color ink) in one task and from conflicting perceptual information (stimulus and response position) in the other task. Therefore, our investigation of the Stroop-like and Simon effects will not be affected by a difference in stimulus attributes in the two tasks. In addition, as in previous studies combining Stroop-like and Simon effects^19,40^, we adopted a Stroop-like task in which the conflict concerned two colored stimulus objects. Importantly, however, at variance with previous studies, which adopted a flanker paradigm, we choose a different Stroop-like paradigm, where the conflict, rather than concerning the target and the flanker, concerned two different parts of the target (i.e., square and frame).

We hypothesized that, if Stroop-like and Simon conflicts are caused by distinct mechanisms operating independently and in linear fashion, as the Perceptual Account suggests^2,5^, then, when both conflicts are present, we should observe additivity. In contrast, if the mechanisms causing the two types of conflicts operate in parallel at the same processing stage or share the same processing resources, then the two effects should interact, showing interactivity in the form of sub-additivity or super-additivity. An under-additive interaction would be indicative of (some) parallel processing (for a thorough explanation, see^61^), whereas an over-additive interaction would be indicative of a shared processing stage between the two factors (the kind of interaction that the Decisional Account would predict).

Also, we hypothesized that, if additivity manifested itself, it should occur in conjunction with different time courses of the two effects, as was previously found by Hommel^20^ (Experiment 1) with linguistic rather than perceptual stimuli. That is, we expect that the Stroop-like effect is smallest for fast responses and increases as responses get slower, whereas the Simon effect is largest for fast responses but decreases as responses slow down. Conversely, if an interaction between Stroop-like and Simon effects occurs, then we expect to observe a much more complex pattern of time courses, as shown by previous studies^19,20^ (Experiment 2 and 3, and Experiment 1, respectively). Specifically, an under-additive interaction, which suggests a facilitatory effect, should occur in conjunction with a Simon effect, that, as reaction times become longer, decreases (and/or reverses) with Stroop-like incongruent trials^20^. In contrast, an over-additive interaction, which suggests an inhibitory effect, should occur in conjunction with a Simon effect, that, as reaction times become longer, increases with Stroop-like congruent trials^19^.

**Figure 1:**
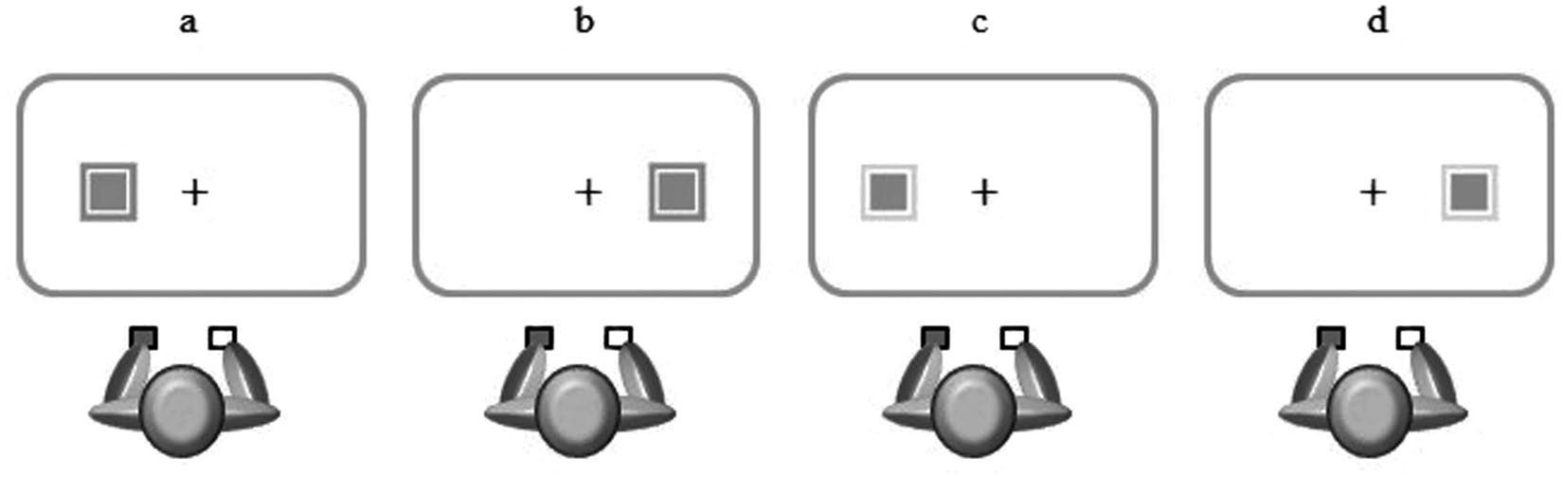
Schematic representation of the stimuli. In this example, instructions required to respond with the left index finger when the stimulus was red and with the right index finger when the stimulus was blue. This two colors are here represented by grey and light grey, respectively. In “panel (a)” the target stimulus is in the Stroop-like congruent and Simon corresponding condition; in “panel (b)” the target stimulus is in the Stroop-like congruent and Simon non-corresponding condition; in “panel (c)” the target stimulus in the Stroop-like incongruent and Simon corresponding; in “panel (d)” the target stimulus is in the Stroop-like incongruent and Simon non-corresponding condition. Note that elements are not drawn to scale.

In Experiment 1, we combined the Stroop-like and Simon tasks. A colored square surrounded by a frame of the same color as the square or of a different color (e.g., “red square - red frame” or “red square - blue frame”; see Fig. 1) was presented on the right or left side of the screen. Possible conflicts were between the square and the frame colors for the Stroop-like task and between square and response positions for the Simon task. In either case, the conflict was perceptual in nature (i.e., two colors; two spatial positions). This experimental design allowed us to downplay, if not to rule out, the contribution of the semantic dimension, to which the differences between Stroop and Simon effects found in previous studies might be attributed. It is also worth noting that previous studies found the classical Stroop effect (with colored words) with either vocal or manual responses. According to Logan and Sbrodoff^62^, studies that compared response modalities often found a stronger Stroop effect with vocal responses than with manual responses^63–70^, though some found no difference between response modalities in the magnitude of the effect^71^.

Experiment 2 was conceived to further investigate the Stroop-like task by combining it with the Simon task. Navon^72,73^ (also see^74^) suggested that perceptual processes could be temporally organized so that they might proceed from the processing of the global structure to more and more fine-grained analysis. Thus, according to Navon’s hypothesis, we should expect faster and more accurate responses if participants are required to discriminate the color of the frame rather than that of the square. If a task is facilitated when the feature to be discriminated pertains to a more peripheral component of the stimulus rather than to a more central one, then one can conclude that the effect ensuing from that task is due to perceptual processing rather than to decisional mechanisms. Thus, we expected that discriminating the color of the frame produced faster and more accurate responses than discriminating the color of the square.

## Results

### Experiment 1

Two participants, who made 16% of errors or more (corresponding to the mean of the errors of all participants plus one standard deviation), were excluded, so that the final sample consisted of 14 participants. Omissions (0.13%) and RTs faster/slower than the overall participant mean minus/plus 2 standard deviations (3.63%) were excluded from the analyses.

Mean Reaction Times (RTs) of correct responses and arcsin-transformed Error Rates (ERs; 7.7% of total trials) were analyzed separately. When sphericity was violated, the Huynh-Feldt correction was applied, although the original degrees of freedom are reported.

To estimate the Stroop-like effect, congruent (i.e., same colors for square and frame) and incongruent (i.e., different colors for square and frame) responses were compared. To measure the Simon effect, corresponding (i.e., the position of the response corresponded to the position of the stimulus) and non-corresponding (i.e., the position of the response did not correspond to the position of the stimulus) responses were compared. The time course of both the RT Stroop-like effect and the RT Simon effect was investigated by applying the Vincentizing procedure^75^. The RT distribution for each participant and congruent/correspondence condition was divided into quartiles (bins), and the mean of RT for each quartile was calculated. We calculated as well the size of both the Stroop-like effect and the Simon effect for each bin, subtracting the mean RT for the congruent responses from the mean RT for the incongruent ones and the mean RT for the corresponding responses from the mean RT for the non-corresponding ones, for the Stroop-like and the Simon effect, respectively.

A repeated-measures ANOVA was run with *Bin* (1–4), *Stroop-like task* (congruent vs. incongruent trials) and *Simon task* (corresponding vs. non-corresponding trials) as within-subjects factors. Note that, considering the way the data were grouped, the *Bin* main effect was necessarily significant in all analyses. Therefore, it is not reported and discussed here or later on. Data are shown in Table 1.

**Table 1:** Mean Response Times (in Milliseconds) and Error Rates (in percentage) for Congruent (CO) and Incongruent (IN), Corresponding (C) and Non-Corresponding (NC) trials, with their 95% Confidence Interval (C.I.) and the resulting Stroop-like and Simon effects.

Experiment		CO	C.I		IN	C.I.		Stroop-like	C	C.I.		NC	C.I		Simon
			Lower	Upper		Lower	Upper			Lower	Upper		Lower	Upper	
Experiment1	RTs	439	417	461	486	458	515	47*	450	422	479	475	450	500	25*
	Ers	6.8	4.5	9.1	9.6	6.8	12.3	2.8	8.4	6.0	10.0	8	5.4	10.5	−0.4
Experiment 2	RTs	451	416	485	498	464	533	47*	465	433	497	484	447	521	19*
	ERs	2.9	1.7	4.0	5.5	3.6	7.2	2.6*	4.2	2.9	5.5	4.1	2.6	5.6	−0.1

The Stroop-like effect is computed by subtracting reactions times and error rates in congruent trials from the ones in incongruent trials, while the Simon effect is computed by subtracting reaction times and error rates in corresponding trials from the ones in non-corresponding trials. Asterisks denote significant differences.

The main effect of *Stroop-like task* was significant, *F*(1,13) = 88.73, *MS*_*e*_ = 1363.603, *p* < 0.001, *η*_*p*_^2^ = 0.872, that is, responses were faster when the square and the frame had a congruent rather than an incongruent color (439 vs. 486 ms). The analysis also revealed a significant main effect of *Simon task*, *F*(1, 13) = 8.509, *MS*_*e*_ = 4130.233, *p* < 0.05, *η*_*p*_^2^ = 0.396, that is, responses were faster when target and response positions were corresponding rather than non-corresponding (450 vs. 475 ms, respectively). The interaction was not significant, *F*(1, 13) = 0.838, *p* = 0. 377, *η*_*p*_^2^ = 0.061, indicating that the *Stroop-like task* and the *Simon task* did not interact and thus yielded additive effects.

**Figure 2:**
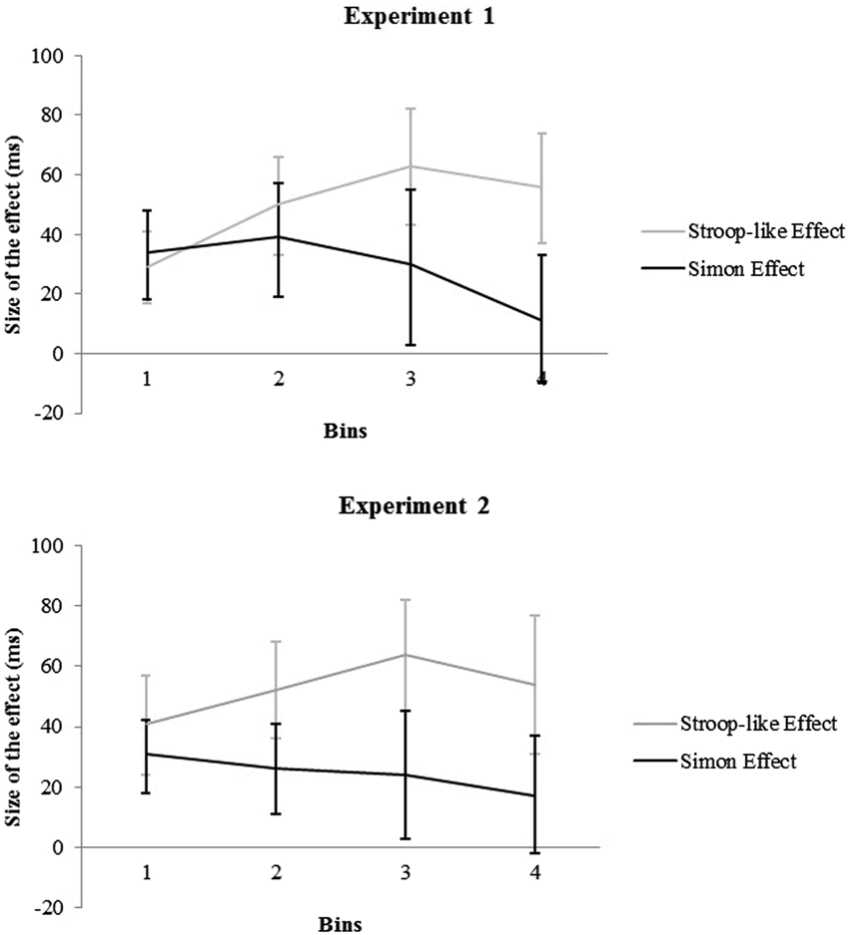
Size of the Stroop-like effect (grey line) and of the Simon effect (black line) as a function of Bins for Experiment 1 (top panel) and Experiment 2 (bottom panel). Bars are confidence intervals.

The *Bin* × *Stroop-like task* interaction was significant, *F*(3, 39) = 17.58, *MS*_*e*_ = 281.498, *p* < 0.001, *η*_*p*_^2^ = 0.575. Paired sample *t-*tests showed that the Stroop-like effect was significant at all bins, *t*_*s*_(13) > 5.258, *p*_*s*_ < 0.001. Helmert contrasts showed that its size increased significantly from bin 1 to bin 3 (29, 50, 63 ms, respectively), *F*_*s*_(1,13) > 8.561, *p*_s_ < 0.012, *η*_*p*_^2^_s_ > 0.397 and remained stable from bin 3 to bin 4 (56 ms), *F*(1,13) = 2.007, *p* = 0.180, *η*_*p*_^2^ = 0.134 (see Fig. 2, top panel).

The *Bin* × *Simon task* interaction was significant too, *F*(3, 39) = 3.802, *MS*_*e*_ = 420.877, *p* = 0.028, *η*_*p*_^2^ = 0.226. Paired sample *t-*tests showed that the Simon effect was significant at bin 1, 2 and 3, *t*_*s*_(13) > 2.474, *p*_*s*_ < 0.028, and not significant at bin 4, *t*(13) = 1.104, *p* = 0.29. Helmert contrasts showed that its size did not change from bin 1 to bin 2 (34 and 39 ms, respectively), *F*(1,13) = 1.523, *p* = 0.239, *η*_*p*_^2^ = 0.105, decreased significantly from bin 2 to bin 3 (30 ms), *F*(1,13) = 15.346, *p* = 0.002, *η*_*p*_^2^ = 0.541, and remained stable from bin 3 to bin 4 (11 ms), *F* (1,13) = 4.227, *p* = 0.060, *η*_*p*_^2^ = 0.245 (see Fig. 2, top panel).

ERs showed no significant main effects or interaction, F_s_ < 4.003, p_s_ > 0.06.

### Experiment 2

Three participants, who made 9% of errors or more (corresponding to the mean of the errors of all participants plus one standard deviation), were excluded, so that the final sample consisted of 16 participants. Omissions (3.46%) and RTs faster/slower than the overall participant mean minus/plus 2 standard deviations (4.16%) were excluded from the analyses.

Mean RTs of correct responses and arcsin-transformed ERs (4.19% of total trials) were analyzed as in Experiment 1. When sphericity was violated, the Huynh-Feldt correction was applied, although the original degrees of freedom are reported.

The main effect of *Stroop-like task* was significant, *F*(1,15) = 86.743, *MS*_*e*_ = 1630.411, *p* < 0.001, *η*_*p*_^2^ = 0.853, that is, responses were faster when the square and the frame had a congruent color rather than an incongruent color (451 vs. 498 ms, respectively). The analysis also revealed a main effect of *Simon task*, *F*(1, 15) = 10.089, *MS*_*e*_ = 2231.946, *p* = 0.006, *η*_*p*_^2^ = 0.402, that is, responses were faster when target and response positions were corresponding rather than non-corresponding (465 vs. 484 ms, respectively). The interaction was not significant, *F*(1, 15) = 1.748, *MS*_*e*_ = 1309.682, *p* = 0.206, *η*_*p*_^2^ = 0.104, indicating that the Stroop-like effect and the Simon effect were additive.

The *Bin* × *Stroop-like task* interaction was significant, *F*(3, 45) = 4.017, *MS*_*e*_ = 429.067, *p* = 0.043, *η*_*p*_^2^ = 0.211. Paired sample *t-*tests showed that the Stroop-like effect was significant at all bins, *t*_*s*_(15) > 5.030, *p*_*s*_ < 0.001. Helmert contrasts showed that its size increased significantly from bin 1 to bin 2 (41 and 52 ms), *F* (1,15) = 5.852, *p* = 0.029, *η*_*p*_^2^ = 0.281 and remained stable from bin 2 to bin 4 (64 and 54 ms), *F*_s_ (1,15) < 1.823, *p*_*s*_ > 0.197, *η*_*p*_^2^_s_ < 0.108 (see Fig. 2, bottom panel).

The *Bin* × *Simon task* interaction was not significant, *F*(3, 45) = 2.276, *p* = 0.135, *η*_*p*_^2^ = 0.132, suggesting a stable Simon effect from bin 1 to bin 4 (31, 26, 24, and 17 ms, respectively), even though, at least numerically, the effect seemed to decline (see Fig. 2, bottom panel).

ERs showed a main effect of *Stroop-like task*, *F*(1, 15) = 4.438, *MS*_*e*_ = 0.16, *p* = 0.05, *η*_*p*_^2^ = 0.228, indicating that responses were more accurate in the congruent than in the incongruent conditions (2.9 vs. 5.5%). No other main effects or interactions were significant, *F*_*s*_ < 0.570, *p*_*s*_ > 0.462, *η*_*p*_^2^_s_< 0.037.

### Additional analyses

Neither Experiment revealed an interaction between Simon and Stroop-like conflicts, thus suggesting additivity between the two effects (see Table 2 for details). However, based on the procedure of null hypothesis significance testing (NHST^76^) the null hypothesis can never be accepted. One just fails to reject it. Therefore, our results are, in a certain sense, still inconclusive. We, therefore, collated the data from participants in both experiments and performed a Bayesian hypothesis testing using the BIC approximation^76,77^. This analysis was aimed at comparing the plausibility of the null and the alternative hypotheses concerning the interaction between Simon and Stroop-like conflicts. It was conducted with the R software program^78^ using the lme4^79^ library. According to Wagenmakers^76^, “assuming the models under consideration are equally plausible a priori, a comparison of their BIC values easily yields an approximation of their posterior probabilities” (p. 796). We found that the BIC approximation of the Bayes factor (BF_01_), expressing the probability of the data given H_0_ (i.e., no interaction) relative to H_1_ (i.e., interaction), was BF_01_ = 5.2 (for a detailed description of how the BIC approximation of the Bayes factor can be derived see Appendix B in^76^; see also^80,81^). Hence, according to the BIC approximation of the Bayes factor (BF_01_), in our experiments H_0_ is between five and six times more likely than H_1_. That strengthens our conclusion in favor of additivity between Simon and Stroop-like conflicts, although additivity emerges from a non-significant interaction.

**Table 2:** Means for each experimental condition: Incongruent (IN)-Non Corresponding (NC), Congruent (CO)-Non Corresponding (NC), Incongruent (IN)-Corresponding (C), and Congruent (CO)-Corresponding (C) with their 95% Confidence Interval (C.I.) for both RTs (*Milliseconds*) and ERs (%) from both Experiment 1 and 2.

Experiment		Stroop	Simon	Mean	C.I	
					Lower Bound	Upper Bound
Experiment 1	RTs	IN	NC	497	469	526
		CO	NC	453	431	475
		IN	C	475	442	508
		CO	C	425	400	451
	ERs	IN	NC	9.2	5.7	12.7
		CO	NC	6.6	4.0	9.3
		IN	C	9.8	6.1	13.6
		CO	C	7.0	4.1	9.9
Experiment 2	RTs	IN	NC	505	465	545
		CO	NC	463	428	499
		IN	C	492	462	522
		CO	C	438	403	473
	ERs	IN	NC	5.3	3.1	7.4
		CO	NC	3.0	1.2	4.8
		IN	C	5.6	3.3	7.9
		CO	C	2.8	1.8	3.7

We also ran a between experiments analysis for both RTs and Ers, which was aimed at verifying our predictions that faster and more accurate responses should be observed when people are required to discriminate the color of the frame. This is because, perceptual processes could be temporally organized such that they might proceed from processing the global structure to more fine-grained analysis^72–74^. The finding of faster RT and more accurate responses when the feature to be discriminated pertains to a more peripheral component of the stimulus (i.e. the frame) would suggest that the processing stage involved is perceptual rather than decisional in nature. Indeed, Navon’s hypothesis specifically concerns perceptual processes.

#### RTs

An ANOVA with *Bin* (1–4), *Stroop-like task* (congruent vs. incongruent trials) and *Simon task* (corresponding vs. non-corresponding trials) as within-subject factors and *Experiment* (1 vs. 2) as between-subjects factor was run. Results showed a marginally significant three-way interaction between *Bin*, *Stroop-like task* and *Experiment*, F(3,84) = 2.712, MS_e_ = 197.311, p = 0.05, *η*_*p*_^2^ = 0.088. While in Experiment 1 Helmert contrasts showed that the size of the Stroop-like effect increased significantly from bin 1 to bin 3 and remained stable from bin 3 to bin 4, in Experiment 2, Helmert contrasts showed that the size of the Stroop-like effect increased significantly from bin 1 to bin 2 and remained stable from bin 2 to bin 4. No other interaction was significant, *F*_*s*_ < 0.785, *p*_*s*_ > 0.506, *η*_*p*_^2^ < 0.027.

#### ERs

An ANOVA with *Stroop-like task* (congruent vs. incongruent trials) and *Simon task* (corresponding vs. non-corresponding trials) as within-subject factors and *Experiment* (1 vs. 2) as between-subjects factor was run. Results showed a main effect of *Experiment*, F(1, 28) = 17.797, MS_e_ = 0.015, p < 0.001, *η*_*p*_^2^ = 0.389, that is, mean percentage of ERs in Experiment 1 was greater than mean percentage of ERs in Experiment 2 (7.7% vs. 4.2%, respectively). No other interaction was significant, *F*_*s*_ < 0.075, *p*_*s*_ > 0.786, *η*_*p*_^2^_s_< 0.003.

## General Discussion

The present study examined the Stroop-like conflict in order to shed light on two different views that attempt to explain it. The Perceptual Account^1–5^ claims that the Stroop-like effect is due to S-S congruence or lack of it. The Decisional Account^49–53^ argues that the Stroop-like effect results from the same mechanisms underlying the Simon effect.

Experiment 1 tested whether discriminating left or right-located colored squares surrounded by a frame of the same color as the square or of a different color (e.g., “red square - red frame” or “red square - blue frame”), yielded additive or interactive Stroop-like and Simon effects. Experiment 2 further investigated whether discriminating the color of the frame rather than that of the square entailed the same or different results compared to Experiment 1. According to Navon’s^72–74^ hypothesis, perceptual processes proceed from the global structure to a more fine-grained analysis of the stimulus. We hypothesized that, if discriminating a characteristic of a more peripheral component of the stimulus facilitates performance, then the experimental manipulation from the task is likely to affect perceptual processing (i.e., stimulus identification stage) rather than decisional mechanisms (i.e., response selection stage).

We found that the Stroop-like and Simon effects were additive in both experiments, which, according to Sternberg’s^24^ AFM, indicates that the processing of the S-S conflict and the processing of the S-R conflict are independent. A Bayesian analysis further confirmed this conclusion by showing positive evidence in favor of the null hypothesis, which attested additivity. In addition, in line with previous studies comparing the distributional properties of Stroop and Simon effects^20^ (Experiment 1; see also^82^ for a discussion on delta plots), the analyses on RT distributions showed different patterns for Stroop-like and Simon effects. In either experiment, the Stroop-like effect was smallest for fast responses and increased as responses slowed down. By contrast, in Experiment 1, and only numerically in Experiment 2, the Simon effect was largest for fast responses and decreased as responses slowed down. Importantly, these results were obtained with perceptual rather than linguistic stimuli (or, at the very least, stimuli that were less prominently linguistic in nature). Indeed, our experiments eliminated, or much attenuated, the confound between stimulus attributes (e.g., semantic vs. non-semantic in nature), which rendered interpretation of the results of previous studies uncertain. Stroop-like and Simon tasks in either experiment involved the conflict of perceptual information: square and frame colors (Experiment 1), and frame and square colors (Experiment 2) for the Stroop-like task and square and response positions (Experiment 1), and frame and response position (Experiment 2) for the Simon task. Therefore, both conflicts in either task concerned non-semantic perceptual information (i.e., color or position). The fact that our Stroop-like effect apparently was not reduced or absent further supports the notion that our stimuli were non-semantic (or less semantic) in nature. Indeed, the Stroop effect with colored words (i.e., the classical Stroop effect) is typically reduced^62,83^ or even absent^65,84^ with manual responses.

The additional analysis performed in order to compare Experiments 1 and 2 revealed that participants made fewer errors when they had to discriminate the color of the frame (Experiment 2) rather than that of the square (Experiment 1). Also, in Experiment 2, participants made fewer errors when they had to discriminate the color of the frame in the congruent condition. That is consistent with the notion that, if a task requires discriminating a characteristic pertaining to a more peripheral component of the stimulus, performance is facilitated.

It is important to note that, rather than adopting a flanker paradigm, as some of the previous studies combining the Stroop and Simon tasks had done^19,40^ we chose a Stroop-like paradigm, in which the target conveys both the relevant (the square color in Experiment 1 and the frame color in Experiment 2) and irrelevant (the frame color in Experiment 1 and the square color in Experiment 2) information. Therefore, we can rule out an explanation based on perceptual grouping (when target and flanker are of the same color) or referential coding (when target and flanker are of different colors)^57–60^.

Our results, besides being generally in favor of the Perceptual Account, support the Dimensional Overlap model^2–4^ and the resulting taxonomy that differentiates Stroop-like from Simon effects. This model assumes that, since the stimuli are unrelated to the responses, no response activation and response competition processes, able to produce compatibility effects, can occur in Stroop-like tasks.

It is worth emphasizing that although De Houwer^49^ showed that the Stroop-like effect is partly due to S-R compatibility based on short-term associations created on the basis of task instructions, he concurrently found an important role of S-S congruence in the occurrence of the Stroop-like effect. He compared word triads in three conditions: identical trials (i.e., trials in which all three words were the same, e.g., blue-*blue*-blue); same-response trials (i.e., trials in which the irrelevant flanker words differed from the middle target word but all three words were assigned to the same response, e.g., purple-*blue*-purple); and different-response trials (i.e., trials in which the irrelevant flanker words differed from the middle target word and were assigned to different responses, e.g., green-*blue*-green). The target word was always the middle word, which in here is reported in italics. Importantly, he mapped two words (e.g., blue and purple) to each response (e.g., left). Participants were asked to respond on the basis of the meaning (Experiment 1) or of the ink color (Experiment 2) of the middle target word. It was shown that same response trials were significantly faster than different-response trials, thus indicating S-R compatibility underlying the Stroop-like effect given that same- and -different-response trials only differed as to whether the flanker and target words were assigned to the same or to different responses through task instructions. However, same-response trials were significantly slower than identical trials, hence suggesting a concurrent impact of S-S congruence on the Stroop-like effect. That is because in both conditions (identical and same-response trials) flanker words were assigned to the same-response as target words, yet target and flanker words only matched on identical trials (e.g., blue-*blue*-blue).

In conclusion, our findings support the Perceptual Account of the Stroop-like effect demonstrating that it is largely due to perceptual processing as shown by additivity, different time courses of the two effects (Stroop-like and Simon effects) and a somewhat facilitated performance in discriminating a characteristic pertaining to a more peripheral component of the stimulus.

## Methods

### Data Availability

The datasets generated and analysed during the current study are available in the Open Science Framework repository, https://osf.io/8nwdz/?view_only = 2527d67247dc4cbeb9ac7ecc8110eed0.

#### Participants

Sixteen students (11 females; 2 left-handed; mean age: 20.53, SD: 1.58) and nineteen different students (11 females; 3 left handed; mean age: 21.26, SD: 3.38) from the University of Bologna participated in Experiment 1 and 2, respectively in exchange for course credits. Participants had normal or corrected-to-normal vision and were naïve as to the purpose of the experiment. The experiment was performed with approval of the ethical committee of the University of Bologna and in accordance with the 1964 Helsinki declaration and its later amendments or comparable ethical standards. Written informed consent was obtained from all individual participants included in the study.

#### Apparatus, stimuli and procedure

The experiments were conducted in a quiet room, where the light was dimmed. Stimuli were presented on a 17 inches video monitor (1.6 Ghz refresh rate) on a white background. The viewing distance was 60 cm. Stimuli presentation and response collection were controlled by E-Prime Professional v2.0 software (http://www.pstnet.com). Stimuli were blue/red squares (visual angle: 1.9° × 1.9°) presented at the left/right of the central fixation cross (0.8° × 0.8°). The squares were always surrounded by a frame that could be either of the same color as the square or of a different color. The colored surface areas covered by the square and by the frame measured approximately the same area (4 cm²; 3.85 cm², respectively). Square and frame of the same color produced the Stroop-like congruent condition, whereas when they were of different colors they produced the Stroop-like incongruent condition.

Trials began with presentation of the fixation cross. After 1000 ms the target stimulus appeared and remained on the screen for 300 ms. Target offset was followed by a blank interval of 2000 ms.

Half of the participants were instructed to press the right key (i.e., “-”) in response to the blue square and the left key (i.e., “z”) in response to the red square. The other participants were assigned to the reverse mapping. They were required to ignore the location of the stimulus and respond only to the color of the square as quickly and as accurately as possible.

The experiments consisted of two blocks of 120 randomly mixed trials, equally distributed across the 8 types of trials (2 Square Colors × 2 Frame Colors × 2 Target Positions) and lasted approximately 10 minutes each. The experimental sessions were preceded by a practice session composed of 24 trials.

Trials were classified into 4 different conditions (Stroop-like congruent and Simon corresponding; Stroop-like congruent and Simon non-corresponding; Stroop-like incongruent and Simon corresponding; Stroop-like incongruent and Simon non-corresponding) based on the presence and nature of the conflict (See Fig. 1 for a schematic representation of the stimuli).

In Experiment 2, the apparatus, stimuli, and procedure were the same as those in Experiment 1. The only difference was that participants were required to respond to the color of the frame (rather than that of the square).
